# Convertible metal-backed glenoid in total shoulder arthroplasty: a single-centre case series with minimum 15 years’ follow-up

**DOI:** 10.1302/2633-1462.61.BJO-2024-0118.R1

**Published:** 2025-01-14

**Authors:** Riccardo Ranieri, Mario Borroni, Giacomo Delle Rose, Marco Conti, Raffaele Garofalo, Alessandro Castagna

**Affiliations:** 1 Department of Biomedical Sciences, Humanitas University, Pieve Emanuele, Milan, Italy; 2 Department of Orthopaedic and Traumatology Surgery, Shoulder and Elbow Unit, IRCCS Humanitas Research Hospital, Rozzano, Milan, Italy; 3 Department of Orthopaedic and Trauma Surgery, Shoulder and Sport Medicine Unit, Miulli Hospital, Acquaviva delle Fonti, Italy

**Keywords:** Total shoulder arthroplasty, Metal-backed glenoid, Complications, Revision surgery, Long-term follow-up, Polyethylene wear, Conversion reverse arthroplasty, glenoids, metal, total shoulder arthroplasty, revision surgery, RSA, soft-tissue, polyethylene wear, reverse shoulder arthroplasty (RSA), Survival analysis, visual analogue scale (VAS) for pain

## Abstract

**Aims:**

The aim of this study was to report long-term clinical outcomes of a modern convertible metal-backed glenoid (MBG) in total shoulder arthroplasty (TSA).

**Methods:**

After a minimum of 15 years, a previously studied cohort of 35 patients who received a modern convertible MBG during the period 1996 to 2005 was contacted for clinical and radiological follow-up. At last follow-up, patients were evaluated radiologically and clinically according to the Constant Score, Simple Shoulder Test, and visual analogue scale for pain. Complications and revisions were recorded, and survival analysis was performed.

**Results:**

At the last follow-up, 20 patients were contacted. Of these, 15 patients had experienced at least one complication, and ten underwent revision surgery. The mean time to revision was 13.8 years (7 to 20). Cuff failure was the most common complication. Conversion to reverse shoulder arthroplasty, while maintaining the baseplate, was possible in five cases, with good results. In patients in whom the baseplate was removed, revision was performed significantly later (18.4 vs 11.1 years; p = 0.016). The general revision-free survival was 73% (95% CI 49.5 to 87.3) at 15 years and 38% (95% CI 11.8% to 64.3%) at 20 years, while MBG revision-free survival was 96.0% (95% CI 74.8% to 99.4%) at 15 years and 54% (95% CI 16.2% to 80.8%) at 20 years. Clinical scores showed a negative trend over time, although not statistically significant. Radiologically, polyethylene wear was observed in all cases and was complete in 12 out of 19 cases, and five glenoids were ‘at risk’ for loosening.

**Conclusion:**

At long-term follow-up, convertible MBG-TSA revealed a high rate of complications and revision surgery, mainly due to soft-tissue failure and polyethylene wear occurring with time. Prompt conversion to RSA maintaining the baseplate provided good results and a low complication rate. Radiological follow-up at about ten years is strictly recommended and, if metal-to-metal contact is observed, conversion to RSA is advisable. These results emphasize the need for continued research into improving TSA outcomes, especially in cases of MBG usage.

Cite this article: *Bone Jt Open* 2025;6(1):82–92.

## Introduction

Since the introduction of glenoid arthroplasty in the first-generation total shoulder arthroplasty (TSA) by Neer et al,^1^ TSA has shown to be a good solution for several degenerative shoulder pathologies. Cemented all-polyethylene (PE) glenoids have become the gold standard and the most commonly used solution due to their low revision rate, with some authors reporting a survival rate of over 80% at 15 and 20 years.^2-7^

However, some unsolved problems remain with cemented all-PE glenoids. At long-term follow-up, radiolucent lines (RLLs) and radiological loosening are reported at a rates of 60% to 100% and 9% to 86%, respectively.^2,4-16^ In large studies, they are associated with decreased functional results and patients dissatisfaction.^7,15-19,19,20^ Rotator cuff failure is another significant concern in TSA: at long-term follow-up, superior migration is reported at rates between 13% and 86%, and is often associated with radiological failures.^2,4-8,10-16,21,22^ Young et al^20^ reported a survival free of secondary cuff dysfunction of 45% at 15 years, with a negative impact on clinical and radiological results. For these reasons, some authors noted that rate of revision may underestimate the rate of failure and unsatisfactory result after TSA.^19^ Indeed, since revision of a failed cemented PE is associated with very high complication rates,^23-25^ surgeons may not be inclined to suggest a revision procedure.

With the goal of improving glenoid fixation and reducing RLL, metal-backed glenoid (MBG) for primary TSA was introduced by Cofield and Daly.^26^ After initial enthusiasm for these implants due to favourable preliminary results, several implants were introduced with different characteristics and designs, but mid- and long-term results were disappointing for most of these new glenoids, leading to general scepticism regarding MBGs.^27-35^ Modern MBGs, with different designs, have shown better results at short- to mid-term follow-up compared to older designs,^36,37^ though long-term results are still lacking. Furthermore, some of them can be converted to reverse shoulder arthroplasty (RSA) without exchanging the baseplate in case of revision;^38,39^ considering the significant rate of soft-tissue failure after TSA,^20^ this feature could potentially simplify several revision procedures.

In 2010, we published the mid-term results of a new convertible MBG in 35 consecutive patients.^28^ No loosening, PE-glenoid disassembly, or other implant-related complications were reported. All functional scores significantly improved after surgery.

The aim of the current study was to evaluate the clinical and radiological outcomes of the same cohort after a minimum of 15 years, and to document rates of prosthetic survival and complications. The hypothesis was that clinical results would improve postoperatively and then deteriorate over time, in conjunction with radiological signs of component wear and soft-tissue disorders.

## Methods

The original report included a consecutive series of 35 patients followed for a mean of 75.4 months (48 to 154) who underwent TSA with a new design of MBG component (SMR System; Lima Corporate, Italy), implanted between 1996 and 2005.^28^ The study was conducted in accordance with the Declaration of Helsinki.^40^ All patients provided informed consent to be involved in the study, and to be contacted for future clinical examination at time of the mid-term study.^28^

All 35 patients from the previous study were called for a consultation and radiographs at our institution (IRCCS Humanitas Research Hospital, Italy) at a minimum follow-up of 15 years. Follow-up on an annual basis was not performed. In case of death, the date of death and any data on surgical procedures related to the prosthesis performed at other centres were recorded. Eight patients were lost to follow-up. Seven patients died (none due to causes related to the prosthesis). One patient had a periprosthetic fracture and underwent open reduction and internal fixation (ORIF) with a plate after seven years without revision of the implant.

In total, we were able to contact 20 patients: 15 (75%) females and five (25%) males. Mean age at the time of operation was 55.5 years (30 to 70). The aetiology was rheumatoid arthritis (RA) in three patients (15%), post-traumatic arthritis in four (20%), and idiopathic osteoarthritis (OA) in 13 (65%). One patient was unable to attend for follow-up because he had undergone revision surgery in another hospital; thus, he was excluded for radiological and clinical analysis but provided clinical information.

### Surgical technique

**Fig. 1:**
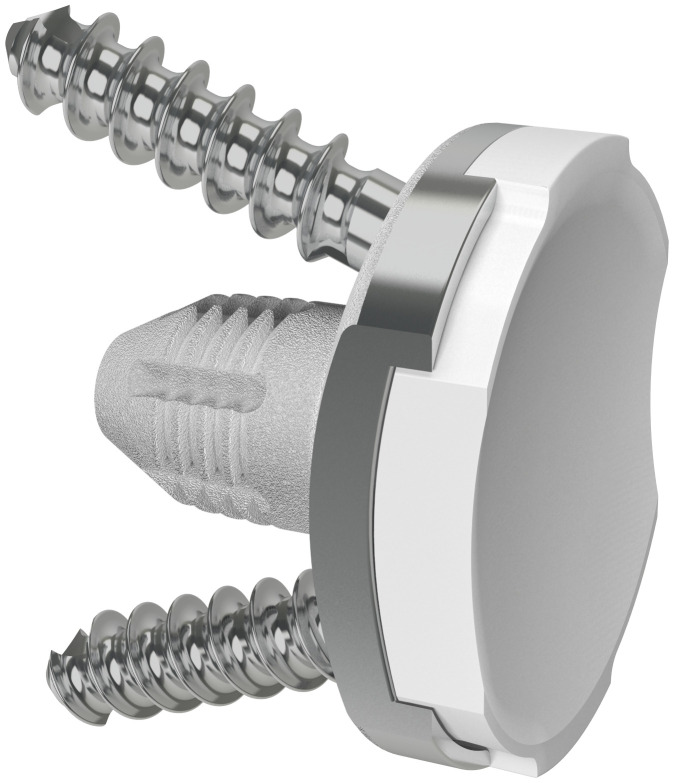
Image of the SMR System glenoid (Lima Corporate, Italy).

A deltopectoral approach was used with a subscapularis tenotomy. A press-fit cementless modular humeral component was used with the cementless MBG component. The glenoid presents a slightly convex back made of a titanium alloy shell and coated with porous titanium and hydroxyapatite (SMR System) (Figure 1). Initial stability is achieved with two 6.5 mm cancellous screws, but the main definitive stability comes from the large hollow central peg. Glenoid-PE surface is concave and the PE surface is non-conforming to reproduce the normal anatomy. Further operative and postoperative details were presented in the original paper.^28^

### Clinical evaluation

The range of motion (forward flexion, external rotation with arm at side (ER1), internal rotation as 0 to 5 score according to the level reached on the back with the thumb), Constant Score (CS),^41^ Simple Shoulder Test (SST),^42^ and a visual analogue scale (VAS) for pain (from 0 = no pain, to 10 = worst possible pain) were recorded on the day before the operation, at mean mid-term follow-up of 75.4 months (48 to 154),^28^ and at the last mean follow-up of 18 years (15 to 25). Strength measurements were carried out using a handheld dynamometer. All patients were analyzed at the last follow-up, even in the case of revision surgery.

### Radiological evaluation

Standardized true anteroposterior radiographs of the shoulders with the humerus in external, neutral, and internal rotation were obtained postoperatively for all patients at the last follow-up of minimum 15 years. Overall, the mean radiological follow-up was 18.1 years (15 to 25). In the case of revision surgery, the same radiographs and/or a CT scan were also performed before the revision. For the radiological analysis of the original prosthesis, the radiograph before revision was used, giving a mean radiological follow-up of 16.1 years (7 to 20).

**Fig. 2:**
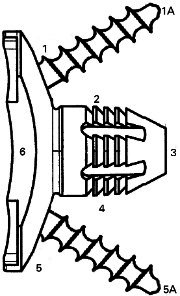
Evaluation of the radiolucent lines. Numbers represent the zones for measurement of the radiolucent lines.

Radiological assessment of the glenoid component was performed with the method described by Mole et al,^43^ and adapted to the shape of this glenoid (Figure 2).^28^ The glenoid was defined to be ‘at risk’ for loosening in case of complete radiolucent line around the glenoid component and if some part of it was ≥ 2 mm, or in case of a change in the component’s position.^44^

PE wear was considered to be present if the thickness between the humeral head and the glenoid tray decreased between the postoperative and latest imaging: it was defined as complete if metal-to-metal contact was observed, or partial if a space between the humeral head and tray component was still visible.^45^

Radiological assessment of the humeral component was performed with the method described by Sperling et al.^44^ The component was divided into eight zones and was defined as being ‘at risk’ if a radiolucent line of ≥ 2 mm wide was present in three or more zones, or if tilt or subsidence of the component were present.

Cranial migration of the humerus was analyzed according to the method of Torchia et al.^15^ It was defined as mild, moderate, or severe if the centre of the prosthetic humeral head had translated less than one-quarter, between one-quarter and one-half, or more than half, respectively, of its diameter relative to the centre of the glenoid component. Moderate or severe subluxation in combination with clinical sign of posterosuperior cuff tear indicated secondary cuff failure.

In the case of conversion to RSA, scapular notching was graded according to the Sirveaux classification.^46^

Two independent observers, a surgeon (RR) and a radiologist not involved with the study, who were blinded to the identity and clinical outcomes of the patients, separately reviewed the radiographs, and discussed their analyses until both had agreed a final score. In the case of revision surgery, both the radiograph before the operation and the radiograph at last follow-up were analyzed.

### Statistical analysis

Survival analysis with general revision (isolated ORIF was not considered as revision) and removal of the glenoid (MBG revision) as an endpoint was carried out using Kaplan-Meier survival curves. For the lost patients and the deceased patients for whom it was impossible to obtain the date of death, the mid-term follow-up was considered as the last follow-up. Numerical outcomes were described as means and SD or range. Discrete outcomes were described as absolute and relative frequencies. The D’Agostino-Pearson test was used to analyze the distribution of the data collected, after which a paired *t*-test or the Mann-Whitney U test was used to evaluate for statistical significance. Qualitative data were compared using the chi-squared test and Fisher’s exact test. The α risk was set at 5%. Statistical analysis was performed with the EasyMedStat software v. 3.20 (EasyMedStat, France).^47^

## Results

### Complications and revisions

**Fig. 3:**
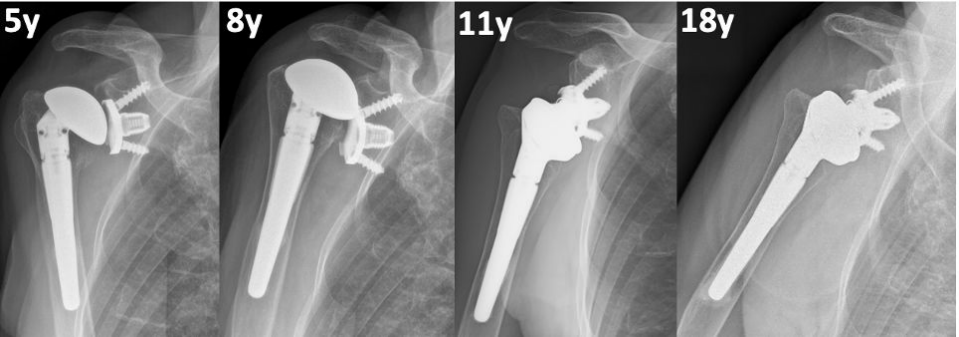
Anteroposterior radiograph views showing a 70-year old female patient who had a traumatic rotator cuff rupture after eight years and underwent conversion to reverse shoulder arthroplasty maintaining the original convertible metal-backed glenoid. After 18 years from the first surgery, the implant is still stable.

**Fig. 4:**
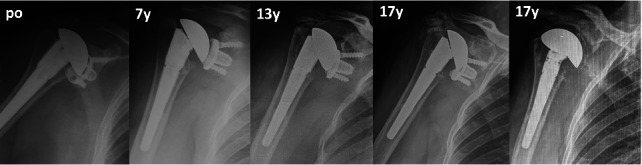
Anteroposterior radiograph views showing a 34-year-old female patient who developed progressive polyethylene eccentric wear with rotator cuff insufficiency noticed at 13 years' follow-up. Conversion to reverse shoulder arthroplasty (RSA) was initially delayed for personal reasons. After 17 years, metal-to-metal contact led to erosion of metal back with severe osteolysis, metallosis with opacification of the periprosthetic soft-tissue (bubble sign^48^ and loosening). Conversion to RSA was not possible, so removal of the baseplate and conversion to hemiarthroplasty was performed. po, postooperative.

**Fig. 5:**
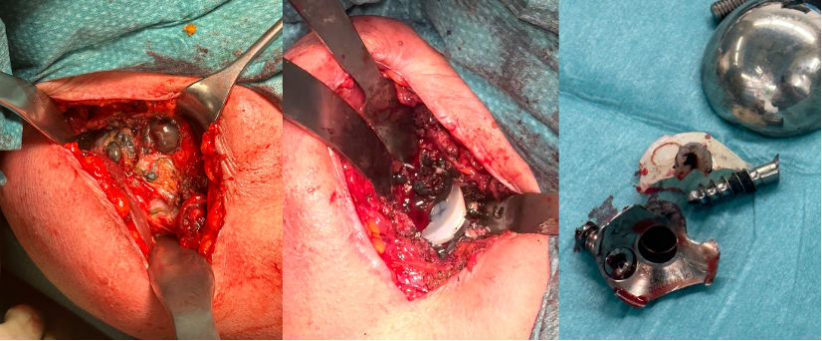
Intraoperative picture of the same case shown in Figure 4. Note the severe eccentric wear of the polyethylene and the metal-backed glenoid, which led to severe metallosis in the surrounding soft-tissue and progressive glenoid loosening.

Overall, at last follow-up, 15 patients had at least one complication, and 11 patients underwent at least one surgical procedure (ten implant revision, two ORIF, one open subscapularis repair). Mean time after any revision was 13.8 years (7 to 20). Ten patients had posterosuperior cuff failure (one following a traumatic dislocation), and of these, six were revised: in four patients it was possible to perform a conversion to RSA without exchanging the MBG (Figure 3); in two cases, glenoid loosening with severe metallosis was present, and a conversion to hemiarthroplasty with cuff tear arthropathy (CTA) head was performed after 17 and 18 years (Figure 4). Three cases had anterosuperior cuff failure with subscapularis insufficiency: of these, one underwent subscapularis repair after eight years and conversion to RSA maintaining the MBG after 16 years, while one patient scheduled for a RSA underwent conversion to hemiarthroplasty with CTA head (despite a stable baseplate, which allowed implantation of the glenopshere, a fracture of the glenoid occurred during reduction manoeuvre because of very weak bone as a consequence of the metallosis). One patient had recurrence of posterior subluxation with glenoid loosening; it was revised to a TSA after nine years with iliac crest bone graft, combined a new SMR AXIOMA TT MBG with a longer peg made of trabecular titanium (AXIOMA; Lima Corporate). After one year, the patient had new recurrence of posterior subluxation with fixed posterior dislocation and glenoid loosening, but refused other surgeries. In all revisions the humeral stem was stable and was not revised; in the case of RSA, it was converted, exchanging only the humeral body. Two patients had a traumatic periprosthetic fracture. One underwent ORIF with plate without implant revision, while the other (operated in another hospital) first underwent ORIF with a plate and, subsequently, due to a second fracture at the same site with breakage of the plate, underwent revision for glenoid and stem removal, implantation of a hemiarthroplasty with a longer stem, and allograft (clinical data regarding this patient were not available). Interestingly, the three patients (two for glenoid loosening and one for glenoid fracture during revision) who underwent hemiarthroplasty with CTA head had at least four years between the evident cuff failure and the revision, developing progressive eccentric poly wear and metal-to-metal contact, with metal erosion, severe metallosis, and compromised bone quality at time of revision (Figure 4 and Figure 5). They undewent revision after a mean of 18.4 years (17 to 20), while patients converted to RSA mantaining the baseplate underwent revision at a mean time of 11.1 years (7 to 17), which was significantly lower (p = 0.016).

Patients who underwent revision surgery had a significantly lower age at last consultation and presented a higher percentage of males compared to patients who did not undergo revision surgery (Table I).

**Table I.** Demographic characteristics of patients categorized according to whether or not they underwent revision sugery.

Variable	Unrevised patients (n = 10)	Revised patients (n = 10)	p-value
Sex (M:F), n	0:10	5:5	0.033*
Mean age at surgery, yrs (SD; range)	61.8 (10.4; 34 to 69)	48.52 (16.2; 30 to 70)	0.121†
Mean age at last consultation, yrs (SD; range)	81.0 (11.9; 50 to 91)	65.9 (15.9: 47.4 to 86)	0.017†
Follow-up, yrs (SD; range)	19.2 (3.3; 16 to 25)	17.4 (1.8; 15 to 20)	0.144†
Diagnosis (FS:OA:RA), n	2:7:1	2:6:2	0.462*
Glenoid (A1:A2:B1:B2), n	5:2:3:0	3:4:1:2	0.410*

* Fisher's exact test.

† Mann-Whitney test.

FS, fracture sequelae; OA, osteoarthritis; RA, rheumatoid arthritis.

### Clinical results

The clinical results of the ten patients who retained the original implant at different follow-up are shown in Table II. There was a negative trend regarding all the clinical scores (except pain) between mid-term and last follow-up, even if the differences were not statistically significant (p > 0.050).

**Table II.** Clinical results at different follow-up for the ten patients who retained the original implant.

Scorse	Preoperative, mean (range)	Mean mid-term, mths (range)	Mean last follow-up, yrs (range)	p-value
Constant Score	33.4 (18 to 48)	69.0 (53 to 83)	57.7 (25 to 83)	0.152*
Simple Shoulder Test	8.3 (6 to11)	4.6 (2 to 6)	5.2 (1 to 10)	0.888†
Visual analogue scale	7.4 (6 to 9)	3.0 (1 to 4)	1.3 (0 to 5)	0.632†
Forward flexion	72 (60 to 90)	131 (90 to 180)	120 (50 to 180)	0.696*
External rotation 1	9 (-10 to 30)	N/A	37 (0 to 80)	N/A
Internal rotation	1.1 (0 to 3)	3.4 (2 to 5)	3.1 (0 to 5)	0.90†

* Student's paired t-test.

† Wilcoxon signed-rank test.

‡ Comparison between last and mid-term follow-up.

N/A, not available.

Clinical results at last follow-up for all the patients, divided according to complications and eventual procedures they underwent following the first operation, are shown in Table III.

**Table III.** Clinical results at last follow-up for all the patients, divided according to the procedure they underwent following the first operation.

Variable	No.	Mean Constant Score (range)	Mean SST (range)	Mean VAS (range)	Mean forward flexion, ° (range)	Mean ER1, ° (range)	Mean IR (range)
No revision – intact cuff	5	75.6 (65.0 to 83.0)	3.0 (1 to 5)	0 (0 to 0)	158° (110 to 180)	62° (40 to 80)	4.4 (3 to 5)
No revision – cuff insufficiency	5	39.8 (25 to 50)	7.4 (6 to 10)	2.6 (1 to 5)	81° (50 to 110)	12° (0 to 20)	1.8 (0 to 4)
Conversion to RSA (mean six yrs (3 to 9) after revision)	6	70.0 (61 to 81)	2.4 (0 to 5)	0.2 (0 to 1)	146° (130 to 170)	20° (10 to 40)	3.6 (2 to 5)
Revision to HA-CTA (mean three mths (2 to 4) after revision)	3	26 (24 to 29)	10 (9 to 11)	3 (0 to 5)	57° (30 to 80)	17° (0 to 40)	1 (1 to 1)
Revision to TSA (six yrs after revision), n	1	41	9	4	100°	20°	1

ER1, external rotation 1; HA-CTA, hemiarthroplasty with cuff tear arthropathy head; IR, internal rotation; RSA, reverse shoulder arthroplasty; TSA, total shoulder arthroplasty; VAS, visual analagoue scale.

### Survival analysis

**Fig. 6:**
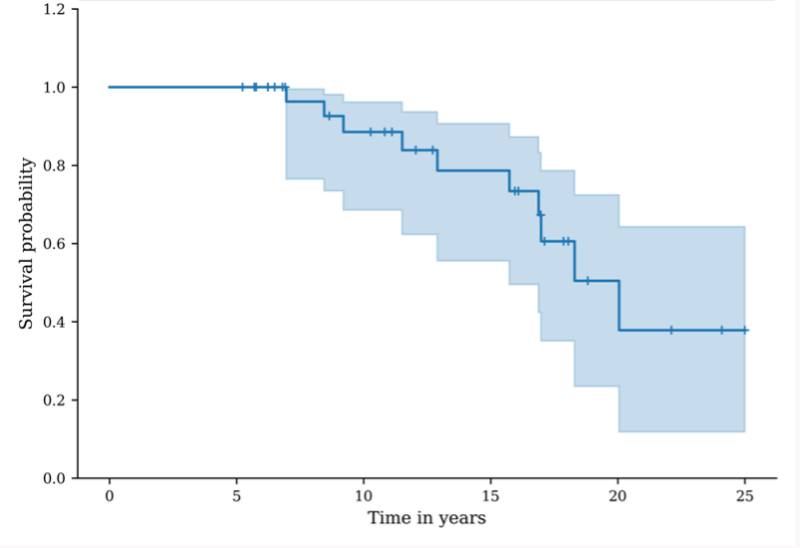
Graph showing survival, considering any revision procedure as the endpoint. The blue line represents the revision-free survival probability, and the shaded area represents the 95% CI.

The general revision-free survival was 100.0% (95% CI 100.0 to 100.0) at five years, 88.6% (95% CI 68.6 to 96.2) at ten years, 73.4% (95% CI 49.5 to 87.3) at 15 years, and 37.9% (95% CI 11.8% to 64.3%) at 20 years (Figure 6).

**Fig. 7:**
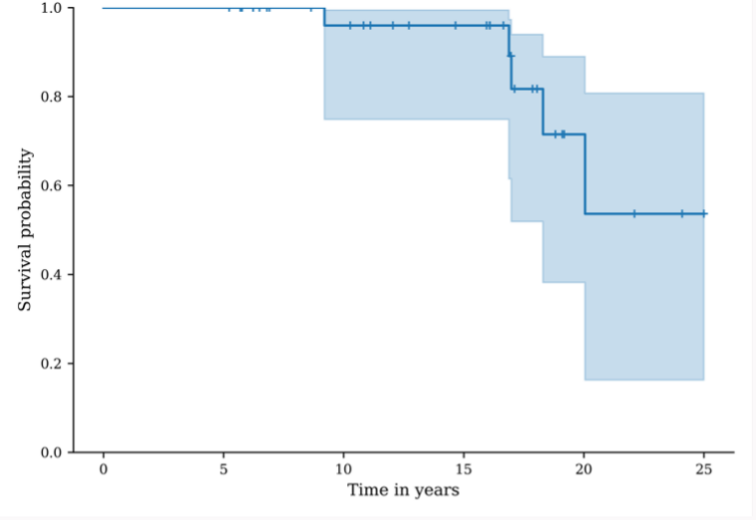
Graph showing survival, considering revision of the metal-backed glenoid as the endpoint. The blue line represents the metal-backed glenoid revision-free survival probability, the shaded area represents the 95% CI.

The MBG revision-free survival was 100.0% (95% CI 100.0 to 100.0) at five years, 96.0% (95% CI 74.8 to 99.4) at ten years, 96.0% (95% CI 74.8% to 99.4%) at 15 years, and 53.6% (95% CI 16.2% to 80.8%) at 20 years (Figure 7).

### Radiological results

At the last follow-up, there were no RLLs around eight glenoid components. Lucent lines incompletely surrounded the glenoid component in six shoulders, while completely surrounding the glenoid in five shoulders. Shoulders with at least one RLL had significantly higher mean radiological follow-up compared to shoulders without any RLL (18.6 years (SD 4.3) vs 12.9 years (SD 4.0); p = 0.009; Student's paired *t*-test). Five glenoid were ‘at risk’ for loosening (Figure 4): three underwent revision and were loose at revision surgery with severe metallosis behind the baseplate.

**Fig. 8:**
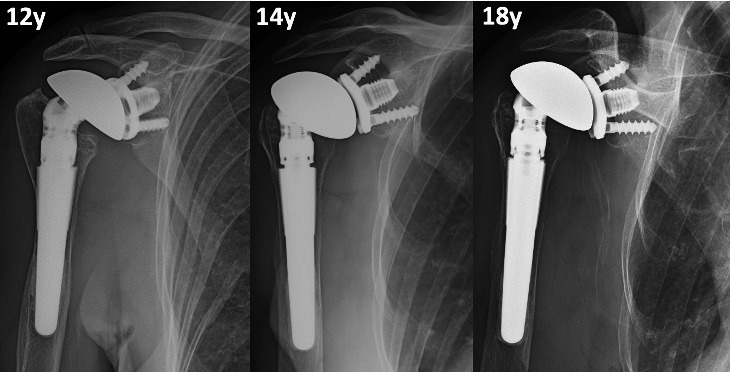
Anteroposterior radiograph views of a 61-year-old female patient with progressive wear of the polyethylene (PE) with reduced thickness between the components. At last follow-up, mild upward migration of the humeral head is observed with complete wear in the superior part of the PE.

All cases presented signs of PE wear: it was partial in seven cases and complete in 12 cases (examples in Figure 4 and Figure 8). Among patients with complete wear and metal-to-metal contact, there was a higher frequency of any RLL (75.0% vs 28.6%; p = 0.074; Fisher's exact test) and glenoid ‘at risk’ (41.67% vs 0.0%; p = 0.106; Fisher's exact test), even if did not achieve statistical significance.

At the last follow-up, excluding zone 8 according to Sperling et al^44^ (where bone resorption often occurs), there were no RLLs around 13 humeral components. Lucent lines incompletely surrounded the proximal humeral component (zones 1 and 7) in four shoulders, while completely surrounding the stem in two shoulders. Two humeral components were defined to be ‘at risk’ (they had 22 years and 25 years of follow-up with severe osteopenia and stress shielding). Shoulders with at least one humeral RLL were significantly more frequent among patients with glenoid at ‘risk’ (80.0% vs 14.3%; p = 0.017; Fisher's exact test).

Upward migration of the humeral head occurred in 17/19 patients (89%): it was mild in five patients, moderate in seven, and severe in five. Among the 12 patients with moderate or severe upward migration, eight underwent revision surgery. Among patients with Torchia moderate or severe migration,^15^ the glenoids considered ‘at risk’ were 5/12 (41.7%), while no glenoid ‘at risk’ was observed in cases of no or mild upward migration (p = 0.106; Fisher's exact test).

Considering patients converted to RSA, at a mean of six years (3 to 9) after the conversion, no patients showed progressive RLLs; four patients presented a scapular notching grade I and one patient had no notching.

## Discussion

This paper presents the results of a convertible MBG at the longest follow-up in the literature. After a mean follow-up of 18 years, half of the available patients underwent revision procedure and 75% presented at least one complication, mostly related to soft-tissue failure. The promising mid-term results were not confirmed at long-term follow-up, while the hypothesis – that clinical results deteriorate over time and component wear combined with soft-tissue disorders occurs – was confirmed.

Cemented all-PE glenoid is recognized as the gold standard; nevertheless, long-term results are controversial. Among different designs, complications have been reported at a rate of between 5% to 35%.^2-16,18,21^ However, considering studies which reported radiological analysis, the radiological cuff failure (moderate or severe proximal migration according to Torchia et al)^15^ is between 24% and 86%, and the glenoid loosening rate is between 9% and 85%.^2,4,4,5,7,8,10-15,21^ These data mean that the definition of complications may vary among different studies, and caution should be used when inferring definitive conclusions. Since glenoid loosening and rotator cuff failure have been shown to negatively affect clinical results in large population studies,^7,15-19^ we considered these findings as complications in our series, reporting up to a 75% rate. Indeed, even if we did not perform a statistical analysis due to low number of cases, unrevised patients with evident cuff tear showed lower functional results in our series.

Long-term studies regarding MBG, analyzing old and non-convertible designs, were extremely disappointing.^22,45,49-51^ The main problem has been shown to be polyethylene wear,^36,52-55^ subsequently leading to metal wear and glenoid loosening, with concomitant soft-tissue disorders. Our long-term results identified the same problems of poly wear and soft-tissue failure as highlighted by Boileau et al^45^ and Taunton et al.^51^ All the patients presented PE wear, which was complete in 12 of 19 cases with metal-to-metal contact. Overall, 13 patients presented rotator cuff failure and one had recurrent posterior instability. However, unlike other studies,^45,51^ the MBG remained stable within the first 15 years (with one exception), allowing a conversion to RSA without changing the baseplate in five cases, simplifying revision from TSA to RSA^38,39,56^ and providing very good results without any complications at six years after surgery. In the three cases where conversion was not possible due to glenoid loosening/fracture, the revision procedure was delayed for several years (because of personal patient-related reasons) and finally performed between 17 and 20 years; severe metallosis with bone and soft-tissue deterioration was observed (Figure 5). Another two cases with a glenoid radiologically at risk presented at 22 and 25 years of follow-up and refused any revisions because they have minimal pain and are of advanced age. This long-term experience prompted us to strongly recommend a radiological follow-up at about ten years: if metal-to-metal contact is observed, annual follow-up should be continued; if patients become symptomatic or signs of advanced metallosis are observed,^57^ conversion to RSA should be recommended without delay.

The scenario of glenoid loosening and cuff failure in the case of an all-PE glenoid is usually different: even if they are associated with lower clinical performance,^7,15-19^ pain is often limited compared to a failed MBG, and in long-term studies revision surgery is often not recommended despite a considerable amount of unsatisfied patients.^5,6,9,11,15,16,22^ Moreover, the revision of a failed all-PE glenoid has shown high rates of complication and re-revision surgery,^23,25,58,59^ limiting the indication for this procedure. All these considerations may explain the overall relatively high survival rate at 15 and 20 years reported for all-PE TSA,^2-7^ despite the considerable rate of component loosening and soft-tissue failures. It is likely that modern improvement in RSA design options (as long screwed peg, augmented baseplate, or custom-made implant) will allow for improved results in revision; however, little evidence is currently available.

Modular MBGs are characterized by a higher revision rate in registry analysis.^60^ However, it should not be underestimated that in the case of revision of this kind of implant, the MBG was retained in 92.8% of the cases,^60^ and the insert was replaced with a glenosphere, a procedure which has been shown to be effective and associated with a low rate of complications.^38,39,56,61^ Our long-term experience reflected registry data: choosing a convertible modular MBG, the higher odds of failure due to component wear and/or soft-tissue failure should be counterbalanced with the benefit of simplifying an eventual revision procedure. However, considering the higher revision rate in younger patients,^60^ and the relevant rate of radiological loosening,^2,4,4,5,7,8,10-15,21^ in long-term studies, even of cemented PE glenoids, a perfect solution regarding glenoid arthroplasty has not yet been found, and the use of a convertible MBG still represents a possible option.

Younger age and male sex were found to be risk factors for revision. Other authors reported a similar association between revision or loosening and younger age^16,18,45,49,60,62^ or male sex,^15,49^ regardless of glenoid fixation method. Clearly, since this category of patients commonly has a higher activity level with subsequently higher joint load, they experience higher rates of component wear, soft-tissue failure, and consequent need for revision surgery. Today, in our practice, we do not recommend glenoid arthroplasty in young and active patients, or, if a TSA with a convertible MBG is performed, we anticipate for the patient very high odds of future surgery, suggesting periodical radiological follow-up and prompt conversion to RSA in case of soft-tissue failure and/or complete PE wear. Another potential solution may be represented by modern hybrid glenoid, but currently only short-term data are available.

Biomechanical studies and review studies underlined PE wear as the main problem of modern MBG because of the mismatch in stiffness between metal, PE, and bone, and subsequently increased stresses in the PE.^52-55^ The PE used in the MBG component of the SMR System is an ultra-high-molecular-weight polyethylene (UHMWPE). In recent years, technology developments have occurred: cross-linked PE (XL-UHMWPE) was introduced in shoulder arthroplasty to decrease wear rates,^63^ and registry data have confirmed a lower rate of revision compared to non-XL-PE glenoid.^60^ Consequently, the application of this technology even in modern MBG may be a future field of innovation, aiming to improve durability and survival rates.

Our radiological analysis showed interesting findings regarding the pattern of failure of this implant. Stable fixation without sign of loosening was obtained with this modern MBG and maintained in the first ten years. However, signs of progressive PE wear and metal-to-metal contact, which was present in all patients at long-term follow-up, led to progressive development of RLLs and glenoid ‘at risk’ appearance. Not by chance, patients presenting with RLLs had longer follow-up (mean 18.6 years) and a higher frequency of complete poly wear. Additionally, humeral RLLs were significantly associated with radiologically loose glenoids. These long-term radiological observations are linked to two possible mechanisms: first, a mechanical one, because progressive component wear and/or soft-tissue disorders create unbalanced forces leading to wear,^64^ micromotion, and a rocking-horse loosening mechanism;^65^ and second, a biological phenomenon occurs with PE and metal debris formation leading to progressive osteolysis around both components.^57,63^ Again, metal-to-metal contact should be strictly monitored after ten years, and ultimate failure of the baseplate should be anticipated, considering conversion to RSA based on the patient’s symptoms and expectations.

Proximal humeral migration was also found to be associated with glenoid loosening, a finding reported by other authors for cemented PE implants,^8,15,16^ and identified as a contributing factor for accelerated PE wear and glenoid loosening via a rocking-horse mechanism.^66^ A biomechanical study performed on the Lima SMR showed that the contact pressures between the rotator cuff and the humeral head were higher than the native shoulder with a possible increase of stresses on the cuff.^65^ It is true that rotator cuff dysfunction is the most common complication in our series, but considering the long follow-up time, this finding may be a consequence of the altered capsular restraints and joint kinematics following shoulder arthroplasty, and of the age-related degenerative changes in the rotator cuff muscles and tendons. Young et al,^20^ with a different implant, reported a survival free of secondary cuff dysfunction of 45% at 15 years, with duration of follow-up as a main risk factor. Moreover, conversion to RSA for rotator cuff was performed after a mean time of 13.8 years.

### Strengths and limitations

The strengths of this study were mainly the follow-up ≥ 15 years for all the patients included in the long-term review, making this study one with the longest follow-up in the literature, and the presence of a radiological evaluation, which allowed for a better understanding of the failure modality of this implant. However, it also presented some limitations. First, 15 patients were lost to follow-up or deceased, and one was able to provide only clinical information by phone. However, considering the long follow-up time, this level of dropout is inevitable. Second, only a small number of patients (n = 35) with different diagnoses were studied, providing weak evidence regarding survival analysis, but our aim was to report specifically the long-term results of the same cohort analyzed in the previous study.^28,67^ Third, as in the previous study, a control group is missing, preventing us from making a direct comparison with other implants.

In conclusion, a gold standard for glenoid arthroplasty in TSA has not yet been reached. This modern convertible MBG showed the ability to solve some problems related to old designs, but a considerable rate of complications and revisions was found at long-term follow-up. The main problem continues to be PE wear and rotator cuff dysfunction occurring with time, while baseplate stability was maintained at 15 years. Conversion to RSA has been shown to be an effective procedure. In case of severe eccentric PE with metal-to-metal contact, strict radiological follow-up is recommended and prompt conversion to RSA is advisable.

**Take home message**

- At 15 years, metal-backed glenoid (MBG) in total shoulder arthroplasty (TSA) revealed a high rate of complications and revision surgery.

- Prompt conversion to reverse shoulder arthroplasty, maintaining the baseplate, provided good results and a low complication rate.

- These results emphasize the need for continued research into improving TSA outcomes, especially in cases of MBG usage.

## Data Availability

The datasets generated and analyzed in the current study are not publicly available due to data protection regulations. Access to data is limited to the researchers who have obtained permission for data processing. Further inquiries can be made to the corresponding author.
